# Genetic differentiation of the regional *Plutella xylostella* populations across the Taiwan Strait based on identification of microsatellite markers

**DOI:** 10.1002/ece3.1850

**Published:** 2015-12-01

**Authors:** Fushi Ke, Shijun You, Weiyi He, Tiansheng Liu, Liette Vasseur, Carl J. Douglas, Minsheng You

**Affiliations:** ^1^ Institute of Applied EcologyFujian Agriculture and Forestry UniversityFuzhou350002China; ^2^ Fujian‐Taiwan Joint Innovative Centre for Ecological Control of Crop PestsFujian Agriculture and Forestry UniversityFuzhou350002China; ^3^ Key Laboratory of Integrated Pest Management for Fujian‐Taiwan CropsMinistry of AgricultureFuzhou350002China; ^4^ Department of BotanyUniversity of British Columbia#3529‐6270 University BoulevardVancouverBritish ColumbiaV6T 1Z4Canada; ^5^ Department of Biological SciencesBrock University500 Glenridge AvenueSt. CatharinesOntarioL2S 3A1Canada

**Keywords:** Air currents, diamondback moth, gene flow, genetic variation, simple sequence repeats

## Abstract

Movement of individuals through events, such as storms or crop transportation, may affect survival and distribution of insect pests, as well as population genetic structure at a regional scale. Understanding what factors contribute to gene flow in pest populations remains very important for sustainable pest management. The diamondback moth (*Plutella xylostella*) is an insect pest well known for its capacity of moving over short to long distances. Here, we used newly isolated microsatellite markers to analyze the genetic structure of nine populations across the Taiwan Strait of China (Taiwan and Fujian). A total of 12,152 simple sequence repeats (SSRs) were initially identified from the *P. xylostella* transcriptome (~94 Mb), with an average of 129 SSRs per Mb. Nine SSRs were validated to be polymorphic markers, and eight were used for this population genetic study. Our results showed that the *P. xylostella* populations could be divided into distinct two clusters, which is likely due to the year‐round airflows in this region. A pattern of isolation by distance among the local populations within Fujian was found, and may be related to vegetable transportation. Considering the complexity of the *P. xylostella* population genetic structure from local and regional to global levels, we propose that developing ecologically sound strategies for managing this pest will require knowledge of the link between behavioral and population ecology and its genetic structure.

## Introduction

The genetic makeup of populations is important in determining their capacity to withstand adverse environments and, if needed, adapt to new conditions (Vignuzzi et al. 2006; Draghi et al. 2010; Hayden et al. 2011; Verhoeven et al. 2011). Population structure and connectivity as well as genetic diversity all define the level of susceptibility of a population and its adaptive capacity to environmental changes (Freeland 2006; Kremer et al. 2012; Pauls et al. 2013). Gene flow, through dispersal and short‐ or long‐distance migration, plays a role in determining genetic variation and evolution of local populations (Alleaume‐Benharira et al. 2006; Kremer et al. 2012; Raymond et al. 2013; Rius and Darling 2014). For insect pests, gene flow can also facilitate population outbreaks and increase the possibility for the spread of insecticide‐resistant genes (Herzig 1995; Margaritopoulos et al. 2009). Different factors, such as the types of human activities, air currents, and climate conditions, as well as the presence of geographic barriers, can facilitate or impede dispersal or migration of insect species (Wei et al. 2013; Niu et al. 2014;). For pest management, understanding how environmental and anthropogenic factors influence individual movements and gene flow is essential at both local and regional levels. Analysis of genetic variation within and among pest populations has been a powerful tool to understand the importance of dispersal or migration and remains an important issue to consider when developing sustainable pest management (Roderick 1996; Raymond et al. 2013).

The diamondback moth (DBM), *Plutella xylostella* (L), represents a typical pest insect that has the capacity to disperse or migrate over short to long distances (Furlong et al. 2013; Philips et al. 2014). This pest of cruciferous species has been successful in adapting to various environmental conditions and has a worldwide distribution (Furlong et al. 2013). Long‐distance dispersal of DBM has been documented and is especially triggered by airflow during desirable meteorological conditions (Chapman et al. 2002; Coulson et al. 2002; Fu et al. 2014). The dynamics of DBM movement at local and regional scales, however, remains less understood and is suggested to be confined primarily to movement between neighboring fields (Mo et al. 2003; Schellhorn et al. 2008).

Population genetic studies of DBM have been carried out, but few examined explicitly the factors influencing regional genetic distribution (Endersby et al. 2006; Li et al. 2006). Wei et al. (2013) report an overall lack of genetic differentiation among all 27 populations analyzed in China, with no correlation between genetic and geographic distances. The annual migration of DBM from southern to northern regions of China may result from strong winds (Fu et al. 2014) and/ or meteorological events (Wei et al. 2013). At the landscape scale, Niu et al. (2014) argue that mountains can shape the genetic structure of DBM populations and vegetable transportation may be responsible for gene flow among local populations. Tabashnik et al. (1987) report significant intra‐island variation in susceptibility to different insecticides among DBM populations of Hawaii and suggest that local factors, such as spraying of conventional insecticides, are probably playing an important role in shaping the genetic structure of DBM populations. These studies suggest that many factors may interact in structuring DBM population genetics. Examining how dispersal or movement mechanisms govern genetic structure and gene flow of this pest can help better understand its ability to rapidly adapt to novel environments.

Fujian and Taiwan are two subtropical provinces on both sides of the Taiwan Strait where vegetable production, including cruciferous plants, is currently intensifying. Both provinces suffer from frequent infestations of *P. xylostella* (Talekar and Shelton 1993; You and Wei 2007). The Taiwan Strait (with an average width of around 200 km between Fujian and Taiwan) is a natural barrier to dispersal of many species (Ge et al. 2012, 2015; Liu et al. 2013). However, it may not be a movement barrier to this herbivore, as it is known to travel a distance of approximately 400–500 km per night (Chapman et al. 2002). Restrictions in vegetable transportation and trade between Fujian and Taiwan may have limited the movement of the species between the two regions. The year‐round monsoons prevailing across Taiwan Strait with important changes of air current directions over the year may also influence gene flow within and among populations of this pest. The objectives of the present study were therefore to: (1) examine the genetic differences of the *P. xylostella* populations from various locations of both sides of the Taiwan Strait and (2) identify the variables governing the dynamics of gene flow and the *P. xylostella* population genetic structure using microsatellite markers.

We used selectively neutral molecular markers to study genetic differentiation in DBM, as they are preferred for studying questions of demographic history as well as gene flow (Cooke and Lees 2004; Meng et al. 2015). From a landscape genetic viewpoint, neutral molecular markers such as simple sequence repeats (SSRs) are optimal in estimating population parameters, because they can give unbiased estimation of genetic diversity, migration rates, and population structure (Manel et al. 2003; Schwartz et al. 2010). High polymorphism and co‐dominance make SSRs suitable for studying populations by not only distinguishing remarkable genetic differentiation, but also providing insights into fine‐scale ecological entities (Roderick 1996; Sunnucks 2000; Selkoe and Toonen 2006). We first isolated effective and neutrally inherited SSR (or microsatellite) markers from the *P. xylostella* transcriptome and then used the polymorphic loci for the genetic analysis of the *P. xylostella* populations collected from both sides of the Taiwan Strait.

## Material and Methods

### Identification of the *Plutella xylostella* SSRs

We downloaded 171,262 nonredundant unigene sequences of the *P. xylostella* transcriptome from the recently published database (DBM‐DB: http://iae.fafu.edu.cn/DBM/) (Tang et al. 2014). Using MIcroSAtellite (MISA) (Thiel et al. 2003), a complete repertoire of SSRs in this dataset was identified with the default settings of motif lengths and minimum repeat numbers, and the incomplete SSRs with a maximum distance of 100 bp between two adjacent complete SSRs. The repeat‐based lengths, and the numbers and frequencies of the complete SSRs are summarized in Table S1 (Supporting information). SSR primers based on the *P. xylostella* transcriptome were then developed using the Primer 3 program (Rozen and Skaletsky 2012) based on flanking sequences.

To identify polymorphic SSRs, we used individuals from three *P. xylostella* strains collected from Fuzhou in China (Fuzhou‐S, 26.08°N, 119.28°E) (You et al. 2013), Nagasaki in Japan (Japan‐S, 32.80°N, 129.92°E), and Wageningen in Netherlands (Netherlands‐S, 52.00°N, 5.40°E). These colonies were maintained on radish seedlings in a greenhouse at 25 ± 1°C with 16 h light/day without exposure to insecticides. These *P. xylostella* samples were individually used for DNA extraction with the DNeasy Blood and Tissue Kit (Qiagen, Hilden, Germany) following the manufacturer's instructions. The relative purity and concentration of the extracted DNA were estimated with NanoDrop ND‐2000 (NanoDrop products, Wilmington, Delaware). The DNA was diluted to a final concentration of 20 ng/*μ*l with double‐distilled water.

Based on a total of 281 primer pairs randomly selected from the *P. xylostella* transcriptome dataset, we performed PCR reactions to validate the effective primer pairs using the extracted DNA of the *P. xylostella* larvae from Fuzhou (Fuzhou‐S). The forward primers of the validated primer pairs were linked with a universal primer *M‐13* (TGT AAA ACG ACG GCC AGT) at their 5′ ends.

We used eight individuals from the three *P. xylostella* strains (three individuals from Fuzhou‐S, two from Japan‐S, and three from Netherlands‐S) to identify polymorphic SSRs. A program developed by Schuelke (2000) for PCR was used with the conditions that the primers contained 10 *μ*M reverse primer, 2 *μ*M forward primer with a tail *M‐13*, and 8 *μ*M fluorescent‐labeled *M‐13*. The temperature conditions were at 94°C for 10 min, and then 36 cycles at 94°C for 30 s, 56°C for 45 s, 72°C for 45 s, followed by 8 cycles at 94°C for 30 s, 53°C for 45 s, 72°C for 45 s, and a final extension at 72°C for 10 min. After testing by agarose gel electrophoresis (AGE), sizes of the amplification were detected with ABI 3730 (Applied Biosystems). GeneMapper 4.1 (Applied Biosystems) was used to assign alleles based on the sizes of PCR amplifications. PCR products with an identical size generated by the same pair of primers were considered as an allele. SSR markers that could steadily produce ≥ 2 alleles among the eight individuals were taken to be polymorphic markers.

### Genetic analysis of the *Plutella xylostella* populations from Fujian and Taiwan

**Figure 1:**
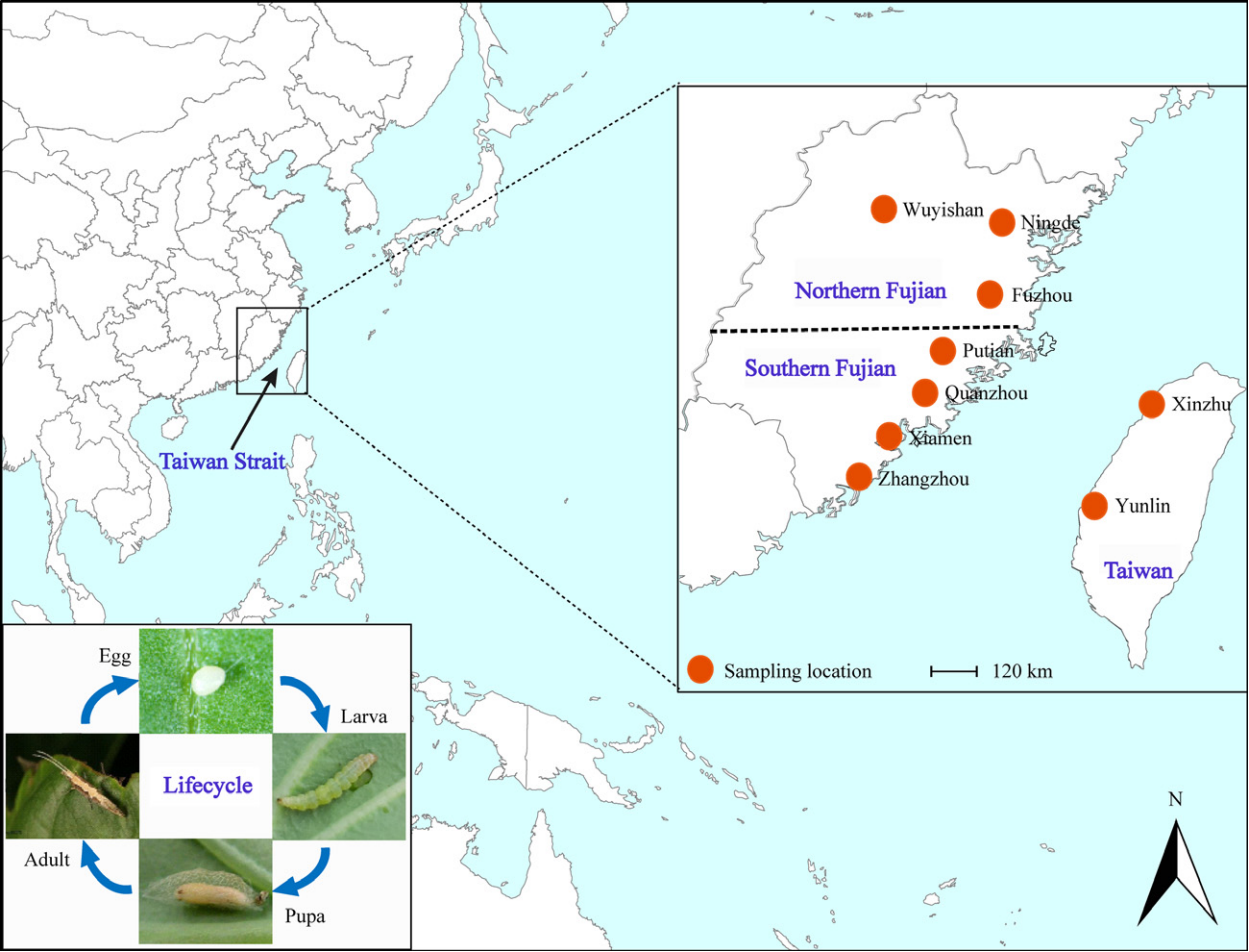
Map showing geographic location of the Taiwan Strait (left) and sampling locations of *Plutella xylostella* used for this study. The inset in bottom left corner shows the life cycle of *P. xylostella*. (Photos by Tiansheng Liu).

A total of 288 individuals were collected from nine locations on both sides (Fujian and Taiwan) of the Taiwan Strait, China (Fig. 1, Table 1). These samples were morphologically checked to confirm their identity and kept at −80°C prior to DNA extraction. Genetic analysis was carried out by assaying genotypes of the previously identified polymorphic SSRs. MICRO‐CHECKER (Van Oosterhout et al. 2004) was used to determine null alleles of each locus and provide the data on corrected allele frequencies. The selective neutrality of the polymorphic SSRs was evaluated by Ewens–Watterson Test using POPGENE 1.31 (Yeh et al. 1997). Deviations from Hardy–Weinberg equilibrium (HWE) at each locus and for each population were calculated and each linkage among polymorphic SSRs was tested with POPGENE 1.31. The observed heterozygosity and expected heterozygosity were calculated for each locus and each population using FSTAT (Goudet 2001). We also calculated allelic richness per population using ADZE‐1.0 (Szpiech et al. 2008), which uses a rarefaction approach to account for differences in sample size. Based on uncorrected and corrected allele frequencies, pairwise genetic differentiations were estimated with *F*_ST_ (Weir and Cockerham 1984), and the significance of differentiation being tested using 10,000 permutation steps with Genepop (Rousset 2008). Similar differentiation pattern was found based on uncorrected and corrected allele frequencies (Table 2).

**Table 1:** Sampling locations, numbers, and collection date of the *Plutella xylostella* (Px) specimens from Fujian and Taiwan, in southeast of China

Region	Sampling location	Geographic coordinates	Px number	Collection date
Northern Fujian	Wuyishan	27.70°N, 118.00°E	16	2012.7
	Ningde	27.13°N, 119.29°E	35	2012.10
	Fuzhou	26.06°N, 119.21°E	42	2011.8
Southern Fujian	Putian	25.52°N, 118.80°E	39	2013.10
	Quanzhou	24.92°N, 118.52°E	37	2013.12
	Xiamen	24.68°N, 118.14°E	32	2014.1
	Zhangzhou	24.04°N, 117.82°E	33	2013.12
Taiwan	Xinzhu	24.91°N, 121.00°E	22	2013.4
	Yunlin	23.72°N, 120.42°E	32	2013.4

**Table 2:** Pairwise differentiation (*F*_ST_) among the *Plutella xylostella* populations sampled from different locations across the Taiwan Strait based on uncorrected (*a*) and corrected (*b*) allele frequencies

Sampled locations	Putian	Zhangzhou	Fuzhou	Ningde	Quanzhou	Wuyishan	Xiamen	Xinzhu
(a) Pairwise differentiation (*F*_*ST*_) based on uncorrected allele frequencies								
Zhangzhou	−0.007							
Fuzhou	***0.032***	***0.039***						
Ningde	***0.045***	***0.047***	***0.012***					
Quanzhou	***0.012***	0.011	***0.051***	***0.064***				
Wuyishan	***0.022***	***0.033***	−0.003	***0.007***	***0.045***			
Xiamen	***0.010***	***0.007***	***0.059***	***0.089***	***0.013***	***0.062***		
Xinzhu	−0.012	−0.005	***0.027***	***0.033***	0.011	0.020	***0.020***	
Yunlin	−0.002	0.004	***0.019***	***0.038***	***0.008***	0.016	***0.013***	−0.003
(b) Pairwise differentiation (*F*_ST_) based on corrected allele frequencies								
Zhangzhou	−0.007							
Fuzhou	***0.029***	***0.038***						
Ningde	***0.041***	***0.044***	***0.006***					
Quanzhou	0.006	0.004	***0.051***	***0.061***				
Wuyishan	***0.022***	***0.034***	−0.003	***0.007***	***0.044***			
Xiamen	***0.010***	0.006	***0.059***	***0.089***	***0.007***	***0.066***		
Xinzhu	−0.012	−0.004	***0.031***	***0.037***	0.005	0.025	***0.019***	
Yunlin	−0.001	0.005	***0.020***	***0.038***	0.001	0.018	***0.013***	−0.002

Numbers in bold italics indicate significant values at *P* < 0.001.

We developed a population‐level phylogeny using neighbor‐joining (NJ) method (Saitou and Nei 1987) in POPTREE2 (Takezaki et al. 2010) based on *D*_*A*_ distance (Nei et al. 1983) with 1000 bootstrap iterations. Principal Coordinates Analysis (PCoA) was performed to visualize the genetic differentiation among the *P. xylostella* populations using the standardized covariance method in GenAlEx 6.5 (Peakall and Smouse 2006) for distance matrix conversion. The population genetic structure and the ancestry proportion of individuals were analyzed using Bayesian clustering method in STRUCTURE (Pritchard et al. 2000) with 500,000 burn‐in and a run length of 500,000 Markov chain Monte Carlo (MCMC) repetitions. Sampling location information was used for assisting the clustering (LOCPRIOR model) (Hubisz et al. 2009). For nine locations across the Taiwan Strait, we started with *K *=* *1, and ran simulations for *K* values of 1 through 9 using 20 independent runs. Log‐likelihood values of each *K* and the rate of change in the log probability of data between successive values of *K* (deltaK) (Evanno et al. 2005) were assessed to determine the optimal genetic clusters using Structure Harvester (Earl and vonHoldt 2012). The optimal genetic clusters were visualized using Distruct (Rosenberg 2004). Hierarchical analyses of molecular variance (AMOVA) among clusters and populations were carried out based on uncorrected allele frequencies using Arlequin 3.01 (Excoffier et al. 2005) to further confirm the population genetic differentiation of the *P. xylostella* populations across the Taiwan Strait. Mantel test for matrix correlation between genetic distance and geographic distance was performed by using IBDWS (Jensen et al. 2005) with 1000 permutations.

We randomly selected three samples representing different geographic locations, Fuzhou (Northern Fujian), Putian (Southern Fujian), and Yunlin (Taiwan), as cases to estimate the migration rate among populations at the regional level. Population size and migration among populations were analyzed based on Bayesian inference using Migrate 3.6.4 (Beerli and Felsenstein 2001; Beerli 2006), which uses a MCMC approach to approximate the posterior of the parameters. Mutation‐scaled population size (*θ*) was estimated by the equation *θ *= 4*Neμ* (where *Ne* is the long‐term (inbreeding) effective population size, *μ* is the mutation rate per site and generation), and the mutation‐scaled migration size (*M*) was estimated by *M *= *m*/*μ* (where *m* is the migration rate per generation). We gradually increased the numbers in Markov chain settings until smooth histograms were observed and modes were within the 50% credibility intervals. The MCMC‐run consisted of a long chain with 5000 recorded steps, 10 concurrent chains (replicates), and 1000 discarded trees per chain. Static heating scheme was also used with four chains of temperature (10,000, 3, 1.5, and 1) with swapping interval of 1.

## Results

### Characterization of the *Plutella xylostella* SSRs

A total of 12,152 SSRs were identified from the *P. xylostella* transcriptome (~94 Mb), with an average of 129 SSRs per Mb. Approximately 95% of the complete SSRs were shorter than 30 bp in length, while less than 0.1% were longer than 50 bp. In terms of the SSR composition, the numbers of motif‐ and length‐specific SSRs were unevenly distributed. Monomers were the most abundant motifs with a frequency of 58.0%, followed by the trimers (26.6%), dimers (12.1%), and tetramers (2.6%) (Table S1, Supporting information).

Based on the PCR validation of 281 primer pairs, 30 pairs of primers produced expected amplicons. High‐quality bands in all of the three *P. xylostella* strains (Fuzhou‐S, Japan‐S, and Netherlands‐S) were generated for 15 SSR loci, among which six were monomorphic and nine polymorphic with trinucleotide repeats (Table 3).

**Table 3:** Characteristics of nine polymorphic SSRs developed in *Plutella xylostella*

Polymorphic SSR	GenBank Accession No.	Motif	Primers (5′‐3′)	*Na*	Observed size (bp)	Unigene /Position in the transcripts/Annotation
*A‐DBM‐16*	KM925133	ATC	F: GTTCGACATCGGCAGAATTT R: TGGAATTTATGTATCAGCCCAA	15	184–238	Unigene34680_All/UTR
*A‐DBM‐133*	KJ701764	CCG	F: TTTAGTGACGAGATGAGCGG R: AGGAATGATGGCAGAAATGG	12	135–177	Unigene99000_All/CDS/Px013469 (unknown function)
*A‐DBM‐142*	KJ701765	TGG	F: GTGCGTCAAATGTCTTGGTG R: CCTATTTGTTGCGGTCCTGT	9	150–174	Unigene26450_All/UTR
*B‐DBM‐1*	KJ701767	AAC	F: CAACAAACACAACGGCAATC R: CTGGTATGTCTCCTGACGCA	8	221–290	Unigene48948_All/CDS/Transcriptional activator cubitus interruptus
*B‐DBM‐23*	KJ701768	CCA	F: TGGCTCCACTCCACAACATA R: CCGTGTCGATGGTTTTGTCT	6	219–234	Unigene145643_All/ CDS/Microtubule‐associated protein futsch
*B‐DBM‐25*	KJ701769	CCA	F: TACAACACCCAACATGCACC R: TGCTTGTCTTGGATACTGCG	8	104–167	Unigene56663_All/ CDS/Microtubule‐associated protein futsch
*B‐DBM‐30*	KM925134	CGC	F: TGCTTATAGCCTCGTAGCCG R: TGAACATCTAGCGGGAGGAC	13	138–177	Unigene113679_All/UTR
*B‐DBM‐34*	KJ701770	CTA	F: CCTCATTTGTCCCATCATCC R: CCGAATGGACGAAAACTGAT	10	131–182	Unigene169897_All/UTR
*B‐DBM‐64*	KJ701771	AAT	F: TCGCCACGATATGTTCGATA R: AGTTGCATTTACAAGCTCCG	7	153–171	Unigene82431_All/UTR

The annotation information is from DBM‐DB (Tang et al. 2014); UTR means untranslated regions; CDS denotes coding sequence. F and R indicate forward and reverse.

### Genetic patterns of the *Plutella xylostella* populations across the Taiwan Strait

Using the nine polymorphic SSR loci, a total of 88 alleles were found in the 288 individuals, with the number of alleles per locus ranging from 6 (*B‐DBM‐23*) to 15 (*A‐DBM‐16*) (Table 3) and an average of 9.78. The observed fixation indexes of all of the identified polymorphic SSRs fell within the 95% confidence interval of theoretical expectation (Table 4), suggesting that the hypothesis for neutral selection could not be rejected for any of these loci. Among the 81 HWE tests performed on the nine SSR loci and nine populations, 27 showed significant deviations from equilibrium (Fisher's method, *P *<* *0.05), but they were not necessarily associated with particular populations and/or loci. Null alleles were detected in 22 of the 81 loci as a result of heterozygote deficiency (showing a significant positive *F*_*IS*_ value, Table 5), 20 of which were associated with HWE deviation. It is likely that the presence of null alleles of each locus was responsible for significant HWE deviations and significantly positive *F*_*IS*_ values (Brookfield 1996; Endersby et al. 2006).

**Table 4:** Analysis for the selective neutrality of the identified polymorphic SSR loci based on Ewens–Watterson Test using POPGENE

Locus	*N* b	OF^c^	Mean^a^	SE^a^^,^ d	*L95* a ^,^ e	*U95* a ^,^ f
*B‐DBM‐34*	576	0.32	0.37	0.02	0.19	0.75
*B‐DBM‐25*	576	0.49	0.44	0.03	0.22	0.82
*B‐DBM‐23*	576	0.51	0.52	0.03	0.26	0.92
*B‐DBM‐30*	576	0.30	0.30	0.01	0.15	0.61
*A‐DBM‐16*	576	0.44	0.26	0.01	0.14	0.51
*B‐DBM‐1*	576	0.59	0.43	0.02	0.22	0.79
*B‐DBM‐64*	576	0.64	0.48	0.03	0.23	0.87
*A‐DBM‐142*	576	0.59	0.40	0.02	0.20	0.75
*A‐DBM‐133*	576	0.23	0.32	0.02	0.17	0.67

a These statistics were calculated using 1000 simulated samples.

b The total number of alleles.

c Observed sum of the square of allelic frequency.

d Standard error of the mean.

e Lower 95% confidence limit.

f Upper 95% confidence limit.

**Table 5:** Genetic diversity at eight microsatellite loci for the sampled *Plutella xylostella* populations across the Taiwan Strait

Loci	Wuyishan		Ningde		Fuzhou		Putian		Quanzhou		Xiamen		Zhangzhou		Xinzhu		Yunlin	
	*H*_o_	*H*_e_	*H*_o_	*H*_e_	*H*_o_	*H*_e_	*H*_o_	*H*_e_	*H*_o_	*H*_e_	*H*_o_	*H*_e_	*H*_o_	*H*_e_	*H*_o_	*H*_e_	*H*_o_	*H*_e_
*B‐DBM‐34*	0.88	0.67	0.63	0.73	0.29	*0.66*	0.38	***0.66***	0.27	***0.64***	0.38	***0.54***	0.45	***0.71***	0.59	0.69	0.63	0.74
*B‐DBM‐23*	0.56	0.54	0.49	0.60	0.36	*0.49*	0.44	0.52	0.27	***0.39***	0.44	0.39	0.45	0.50	0.50	0.52	0.41	0.46
*B‐DBM‐30*	0.69	0.67	0.49	0.58	0.69	0.68	0.51	***0.70***	0.70	0.74	0.69	0.77	0.55	***0.71***	0.64	0.65	0.69	0.72
*A‐DBM‐16*	0.06	0.06	0.06	0.06	0.05	0.05	0.41	***0.69***	0.57	***0.75***	0.44	***0.80***	0.42	***0.72***	0.36	***0.67***	0.56	0.60
*B‐DBM‐1*	0.31	0.42	0.31	0.36	0.48	0.53	0.26	***0.43***	0.30	0.34	0.28	***0.40***	0.39	0.44	0.32	0.36	0.31	0.38
*B‐DBM‐64*	0.31	0.29	0.34	0.34	0.29	0.37	0.26	0.28	0.22	***0.43***	0.31	***0.42***	0.27	0.36	0.32	0.35	0.25	***0.38***
*A‐DBM‐142*	0.31	0.35	0.40	0.38	0.43	0.44	0.38	0.37	0.49	0.48	0.34	0.43	0.36	0.44	0.27	0.24	0.41	0.41
*A‐DBM‐133*	0.88	0.77	0.83	0.75	0.88	0.78	0.64	***0.78***	0.70	0.81	0.53	***0.74***	0.67	0.72	0.68	0.75	0.75	0.77
Mean	0.50	0.47	0.44	0.48	0.43	***0.50***	0.41	***0.55***	0.44	***0.57***	0.43	***0.56***	0.45	***0.58***	0.46	***0.53***	0.50	***0.56***
Total alleles	28		40		52		44		46		46		46		40		45	
Allelic richness	3.50		4.33		5.05		4.91		5.20		5.25		5.07		4.86		5.12	
Specific alleles	2		4		4		3		2		3		2		1		2	

*H*_o_ denotes observed heterozygosity; *H*_e_ refers to expected heterozygosity; *H*_e_ in bold italic indicates a significant positive *Fis* value (heterozygote deficiency) with *P* < 0.05 based on 1440 randomizations.

Our analysis showed that *B‐DBM‐23* and *B‐DBM‐25* exhibited linkage disequilibrium in all nine *P. xylostella* populations. These two loci were located at scaffold 89 in the published DBM genome (You et al. 2013) and encoded the same protein, which implied the underlying mechanism associated with their linkage disequilibrium. We therefore removed *B‐DBM‐25* from the rest of the analyses, meaning that the following analyses were completed on eight SSR loci. Across the different sampled locations, the number of alleles ranged from 28 in Wuyishan to 52 in Fuzhou. The average expected heterozygosity (*H*_e_) ranged from 0.47 in Wuyishan to 0.58 in Zhangzhou, and the allelic richness ranged from 3.50 in Wuyishan to 5.25 in Xiamen. A total of 23 population‐specific alleles were identified (Table 5).

**Figure 2:**
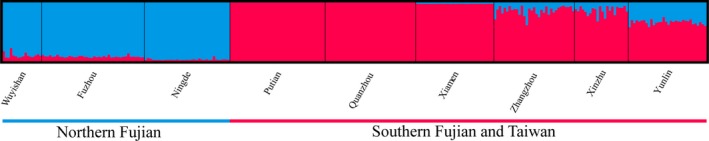
Population structure plot showing two distinct clusters of the *Plutella xylostella* populations sampled from nine different locations across the Taiwan Strait. Individuals are indicated by vertical bars with different colors to denote the membership of location‐associated populations.

**Figure 3:**
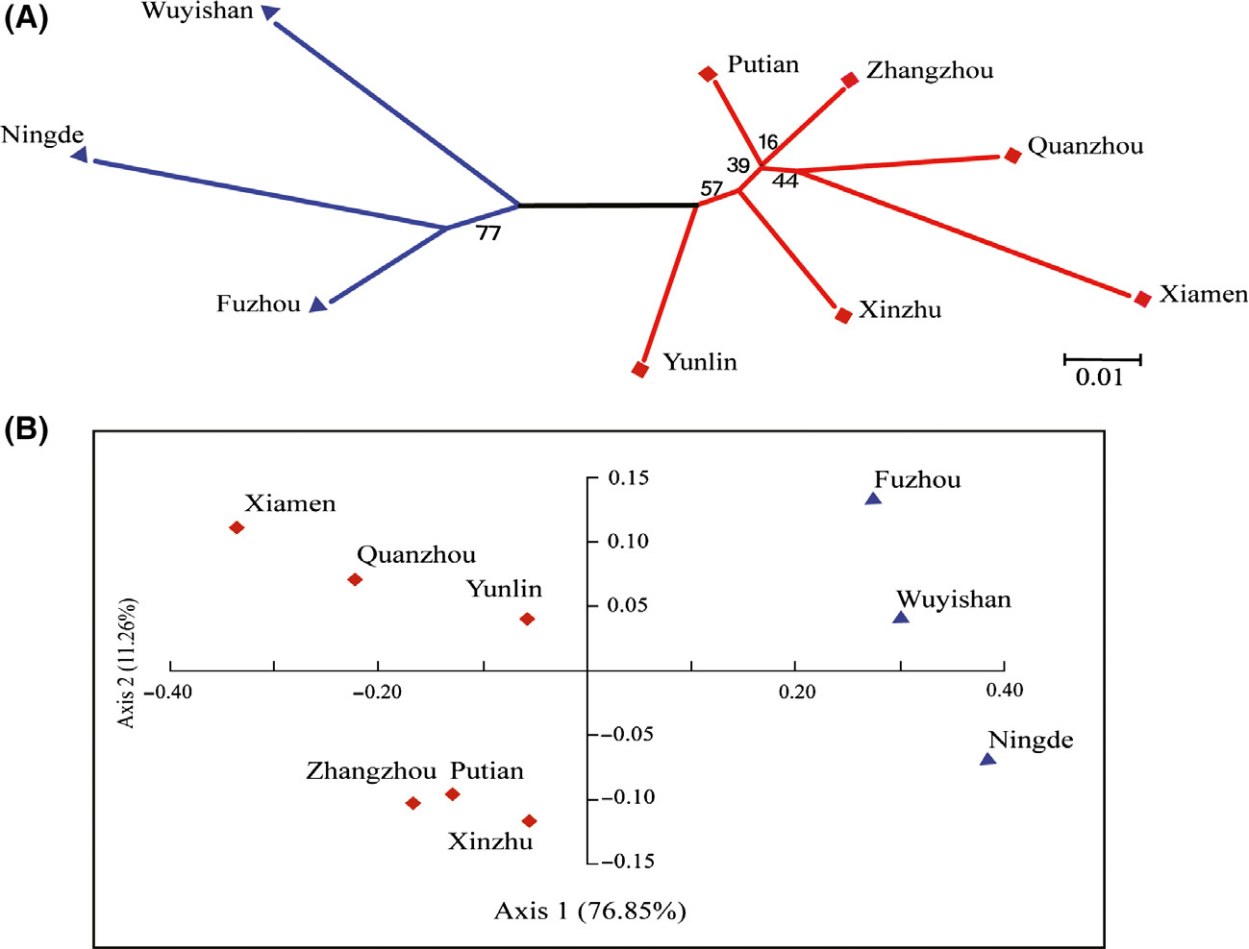
Neighbor‐joining tree based on 1000 bootstraps (A) and Principal Coordinates Analysis (B) of the *Plutella xylostella* populations sampled from different locations in Fujian and Taiwan. Two groups (*K *=* *2) are intuitively clustered with colored triangles and diamonds to indicate the membership of location‐associated populations.

**Figure 4:**
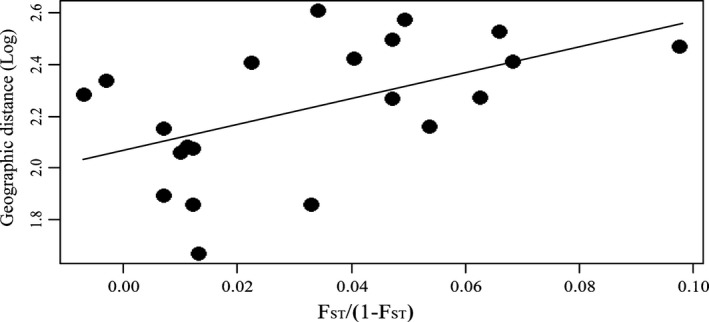
Correlation analysis between the geographic distance (log) and genetic distance (*F*_ST_ /(1−*F*_ST_)) among the *Plutella xylostella* populations sampled from different locations in Fujian province (*R* ^2^ **= 0.271; *P *=* *0.028).

The *P. xylostella* populations across the Taiwan Strait exhibited genetic differentiation among different sampled locations. Based on the Bayesian cluster analysis, the optimal number of clusters was identified to be *K *=* *2 (Fig. S1). Therefore, each of the 288 individuals was proportionally assigned to the two clusters (Fig. 2) composed of (1) South Fujian and Taiwan (including Putian, Quanzhou, Xiamen, Zhangzhou, Xinzhu, and Yunlin), and (2) Northern Fujian (including Wuyishan, Ningde, and Fuzhou). Three‐level hierarchical AMOVA analysis supported the result of Bayesian cluster analysis with two genetic clusters (df = 1, percentage of variation = 3.65%, *P *=* *0.0068). Similar patterns were observed using population‐level phylogenetic analysis (Fig. 3A). These results were further verified through the PCoA analysis (Fig. 3B). Analysis of the *P. xylostella* populations of Fujian showed that the genetic distance significantly increased with the geographic distance (Fig. 4), which indicated that more genetically similar relationships were found for nearby populations than more distant populations.

The pairwise *F*_ST_ values were low to moderate with a maximum between Xiamen and Ningde (0.089), which indicated a high level of movement among populations. The differentiation values between clusters (cluster I vs. cluster II) were generally higher than those within clusters (Table 2). The mutation‐scaled population sizes (*θ*) of the sampled populations were similar, and mutation‐scaled migration rates (*M*) estimated with Migrate showed high gene flow among different geographic regions, with the highest value between Putian (Southern Fujian) and Yunlin (Taiwan) (Table 6).

**Table 6:** Mutation‐scaled population sizes (*θ*) and migration rates (*M*) among the *Plutella xylostella* populations sampled from Fuzhou, Putian, and Yunlin, estimated with Migrate

Parameter	Location		Percentiles		Maximal posterior value	Median
	From	To	2.5%	97.5%		
*θ*		Putian	0.094	0.100	0.099	0.098
*θ*		Fuzhou	0.095	0.100	0.098	0.098
*θ*		Yunlin	0.094	0.100	0.098	0.098
*M*	Fuzhou	Putian	34.000	82.667	59.000	59.667
*M*	Yunlin	Putian	30.000	76.667	53.667	54.333
*M*	Putian	Fuzhou	27.333	70.667	49.667	50.333
*M*	Yunlin	Fuzhou	18.667	62.667	41.000	41.667
*M*	Putian	Yunlin	49.333	100.667	75.000	75.667
*M*	Fuzhou	Yunlin	24.000	80.667	51.000	52.333

## Discussion

### Identification of SSR markers from the *Plutella xylostella* transcriptome

Conventional methods of microsatellite identification from partial genomic libraries have proved to be inefficient for some taxa such as Lepidoptera (Zhang 2004). Low abundance of SSRs, existence of microsatellite DNA families (microsatellite sequences with similar or almost identical flanking region) and polymorphism of the flanking regions, which cause the failure of amplification in Lepidoptera genomes may be associated with low isolation efficiency of SSR markers via traditional laboratory approaches (Ji et al. 2003; Meglecz et al. 2004; Zhang 2004; Meglécz et al. 2007). It is possible to rationally justify the low amplification efficiency by assuming polymorphic flanking regions of microsatellite loci in *P. xylostella*, suggested by the heterozygous nature of the recently published genome of this species (You et al. 2013). Such a hypothetical explanation is supported by observed single nucleotide polymorphisms (SNPs) for several flanking regions of the same microsatellite locus in *P. xylostella* (data not shown). Based on the 281 selected primer pairs, we found that 30 primer pairs could amplify expected sizes in Fuzhou strain, of which 15 SSR loci showed effective bands in all the three *P. xylostella* strains collected from different countries, while others failed and may be related to the polymorphic flanking regions presented in different *P. xylostella* strains.

Microsatellites can be under selection as these repeats may have functions such as regulation of gene activities (Li et al. 2002). The neutrality of microsatellites should therefore be tested before being used in answering ecological questions such as the significance of dispersal (Selkoe and Toonen 2006). No selection was detected in the remaining eight loci, which indicated that these markers were desirable for the analysis of neutral genetic variation in the *P. xylostella* populations.

### Genetic variation of the *Plutella xylostella* populations

Using the eight successfully genotyped polymorphic SSR loci, the initial analysis of the nine populations showed that overall genetic diversity of these *P. xylostella* populations was higher than that of other insect species, such as *Nilaparvata lugens* Stål (Jing et al. 2012) and *Diabrotica virgifera* (Kim et al. 2008) using similar molecular markers. Romiguier et al. (2014) investigated the genetic diversity of 76 nonmodel animal species by sequencing their transcriptomes, and show that short‐lived or highly fecund species are genetically more diverse than the long‐lived or low‐fecundity species with brooding ability. *P. xylostella* is an insect pest with high fecundity and short developmental duration (up to 19 generations per year in Fujian and Taiwan (You and Wei 2007)), which may contribute to this higher population genetic diversity compared with other insect species (Kim et al. 2008; Jing et al. 2012). However, compared with other studies analyzing *P. xylostella* population using genomic SSR loci (Endersby et al. 2006; Wei et al. 2013), our results show low diversity, possibly due to the conservativeness of the SSR markers isolated from the transcriptome (Kim et al. 2008; Wang et al. 2014).

The effectiveness of these polymorphic microsatellite markers in identifying weak but significant genetic structure of *P. xylostella* was important in defining two main clusters among populations across the Taiwan Strait. The first cluster included populations collected from Southern Fujian and Taiwan and the second cluster consisted of populations sampled from Northern Fujian. Despite the fact that the populations were collected at different dates, we believe that these clusters are accurate and independent of collection dates. In Australia and New Zealand, high genetic similarity across the *P. xylostella* populations was found over a couple of years (2001–2003) and could be attributed to gene flows originating from frequent vegetable transportation (Voice and Chapman 2000) and prevailing winds (Endersby et al. 2006). In China, Fu et al. (2014) show, using light‐trapping observations, that movements of *P. xylostella* across Bohai Gulf are consistent over a period of 11 years, most likely contributing to a stable and consistent pattern of gene flow, which was coincident with genetic similarity between populations from Central China and populations from Northeast China evidenced by two independent researches (Wei et al. 2013; Yang et al. 2015). These pieces of evidence indicated that these clusters are not formed based on the collection time.

The genetic similarity among populations of Southern Fujian and Taiwan in our first cluster suggests that airflow across Taiwan Strait might be the main factor for genetic similarity among populations, with dominant winds being southwestward from June to August and northeastward from September to April (Hwang et al. 2006), linking Southern Fujian to Taiwan and vice versa. Such a meteorological pattern favors the formation of genetically similar *P. xylostella* populations in cluster one by homogenizing genetic variation through gene flow. These winds do not connect populations in Northern Fujian with populations in Southern Fujian and Taiwan, which may explain the differentiation of the two clusters.

When our analysis was restricted to the *P. xylostella* populations of the Fujian province, nearby local populations were genetically more similar than populations isolated by longer geographic distances. In addition, while dominant winds across Taiwan Strait did not contribute to gene flow between some of the populations, they showed high genetic similarity (i.e., populations within Southern Fujian) (Fig. 1). We believe that this may be linked to transportation of vegetables and other plant products (Delgado and Cook 2009; Boykin et al. 2010; Niu et al. 2014). In Fujian, majority of agricultural products are supplied by small‐scale farms and usually at the local or regional scale (Rao 2012). Large numbers of rural areas produce their own vegetables and are self‐sufficient. Urban areas such as Xiamen and Fuzhou, however, must import vegetables from various nearby counties, which bring the possibility for the pest to be transported in urban centers, where it also may be mixed. Such conditions allow for gene flow among nearby populations.

At the same time, our results showed that, in the first cluster, genetic diversity within each population was generally higher than in populations of the second cluster. On the contrary, lower numbers of specific alleles were generally found in populations of cluster one when compared with those in cluster two (except population Wuyishan due to small sample size). Gene flow mediated by large‐scale movements can also shape genetic variation within populations (Freeland 2006; Kremer et al. 2012; Raymond et al. 2013; Pierce et al. 2014). Another aspect that should be considered when examining genetic diversity within these two clusters is that both Fujian and Taiwan regions possess year‐round intensive Brassica crop production. The presence of persistent populations of *P. xylostella* at different developmental stages in these regions (You and Wei 2007) can contribute to the accumulation of mutations. New mutations accumulated in local populations and higher levels of dispersal thus may significantly increase genetic diversity in Southern Fujian and Taiwan populations compared with Northern Fujian populations, where gene flow with other regions is relatively low.

## Conclusion

The diamondback moth is an insect pest worldwide distributed, with short‐ to long‐distance dispersal capability. Our analysis shows that several factors can play a role in defining genetic variation and structure at both local and regional levels. Our results support the fundamental role of air currents in intermixing the *P. xylostella* populations from southern Fujian and Taiwan, and that vegetable transportation among rural and urban centers can enhance the complexity of gene flow. In terms of factors affecting population genetic structure at local to regional scales, this complexity may not always be recognized as an important force shaping population genetic diversity of insect pests. Further studies, using landscape genetics and information‐theoretical selection model may help contribute to disentangle the influence of these various mechanisms in governing the gene flow in DBM from local to regional levels.

## Conflict of Interest

None declared.

## Data Accessibility

Microsatellite data file has been accessioned at Dryad (datadryad.org) under the title: “Genetic differentiation of the regional *Plutella xylostella* populations across the Taiwan Strait based on identification of microsatellite markers” (doi:10.5061/dryad.8k3p5).

## Supporting information


**Table S1**. Composition, abundance (number), and frequency of SSRs identified from the *P. xylostella* transcriptome.

**Figure. S1.** Mean (± standard deviation, SD) of log‐likelihood values (A) obtained using the program structure, and delat*K* (B) from 20 independent runs.
