# Supplementary figures and images for: Genetic differentiation of the regional Plutella xylostella populations across the Taiwan Strait based on identification of microsatellite markers
Source: Ecol Evol. 2015 Dec 1;5(24):5880–91. doi: 10.1002/ece3.1850 (PMC4717340; doi:10.1002/ece3.1850)

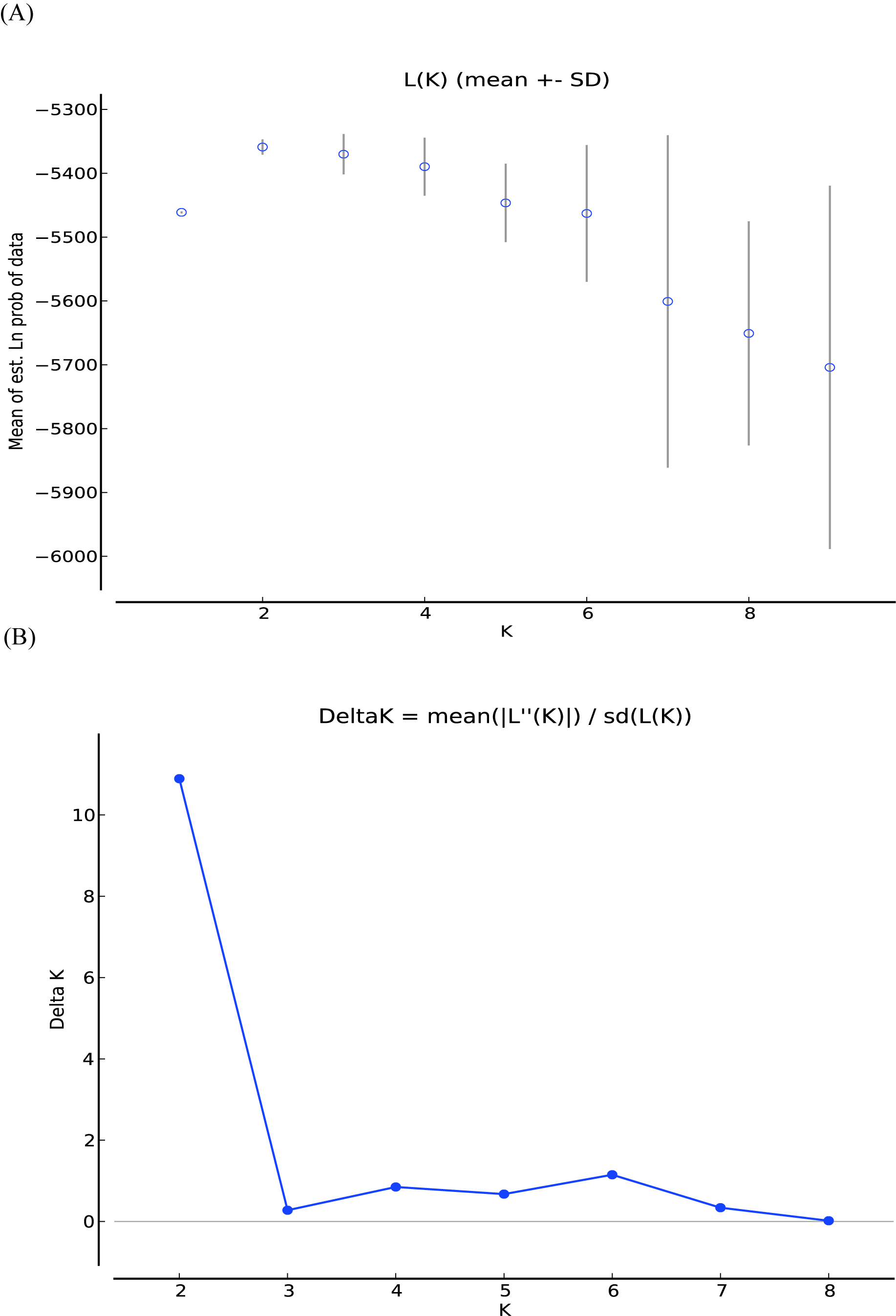

Supplement: Supplementary file 1 — Table S1. Composition, abundance (number), and frequency of SSRs identified from the P. xylostella transcriptome. [file ECE3-5-5880-s001.tif]
