# Supplementary material for: Genetic differentiation of the regional Plutella xylostella populations across the Taiwan Strait based on identification of microsatellite markers
Source: Ecol Evol. 2015 Dec 1;5(24):5880–91. doi: 10.1002/ece3.1850 (PMC4717340; doi:10.1002/ece3.1850)
Supplement: Supplementary file 2 — Figure. S1. Mean (± standard deviation, SD) of log‐likelihood values (A) obtained using the program structure, and delatK (B) from 20 independent runs. [file ECE3-5-5880-s002.doc]

**Table S1 Composition, abundance (number) and frequency of SSRs identified from the *P. xylostella* transcriptome.**

| **SSR** | **Motif** | **Length-specific number of SSRs** | | | | **Total number of SSRs** | **Frequency of SSRs (%)** |
| --- | --- | --- | --- | --- | --- | --- | --- |
|  |  | <30 bp | 30-39 bp | 40-49 bp | ≥50 bp |  |  |
| **Monomer** | A | 2016 | 7 | 2 | 3 | 2028 | 16.7 |
|  | G | 1550 | 9 | 3 | 3 | 1565 | 12.9 |
|  | C | 1445 | 9 | 1 | 1 | 1456 | 12.0 |
|  | T | 1990 | 7 | 3 | 3 | 2003 | 16.5 |
|  | **subtotal** | **7001** | **32** | **9** | **10** | **7052** | **58.0** |
| **Dimer** | AC/CA | 418 | 6 | 5 | 10 | 439 | 3.6 |
|  | AG/GA | 93 | 0 | 0 | 0 | 93 | 0.8 |
|  | AT/TA | 205 | 0 | 0 | 0 | 205 | 1.7 |
|  | CG/GC | 169 | 0 | 0 | 0 | 169 | 1.4 |
|  | CT/TC | 103 | 0 | 1 | 3 | 107 | 0.9 |
|  | GT/TG | 430 | 7 | 5 | 10 | 452 | 3.7 |
|  | **subtotal** | **1418** | **13** | **11** | **23** | **1465** | **12.1** |
| **Trimer** | AAT/ATA/TAA | 218 | 0 | 0 | 0 | 218 | 1.8 |
|  | TTA/TAT/ATT | 203 | 1 | 0 | 0 | 204 | 1.7 |
|  | CCG/CGC/GCC | 647 | 0 | 0 | 0 | 647 | 5.3 |
|  | GGC/GCG/CGG | 627 | 1 | 0 | 0 | 628 | 5.2 |
|  | Others | 1534 | 2 | 1 | 2 | 1539 | 12.7 |
|  | **subtotal** | **3229** | **4** | **1** | **2** | **3236** | **26.6** |
| **Tetramer** | TTTA/TTAT/TATT/ATTT | 1 | 0 | 0 | 0 | 73 | 0.6 |
|  | AAAT/AATA/ATAA/TAAA | 58 | 0 | 0 | 1 | 59 | 0.5 |
|  | Others | 174 | 3 | 2 | 3 | 182 | 1.5 |
|  | **subtotal** | **305** | **3** | **2** | **4** | **314** | **2.6** |
| **Others** | **subtotal** | **66** | **13** | **3** | **3** | **85** | **0.7** |
| **All SSRs** | **Total** | **12019** | **65** | **26** | **42** | **12152** |  |
